# Epidemiology and factors associated with preterm births in multiple pregnancy: a retrospective cohort study

**DOI:** 10.1186/s12884-023-06186-0

**Published:** 2023-12-18

**Authors:** Samita Seetho, Kiattisak Kongwattanakul, Piyamas Saksiriwuttho, Kaewjai Thepsuthammarat

**Affiliations:** 1 Department of Obstetrics and Gynecology, Faculty of Medicine, Khon Kaen University, Khon Kaen, Thailand; 2 Clinical Epidemiology Unit, Faculty of Medicine, Khon Kaen University, Khon Kaen, Thailand

**Keywords:** Epidemiology, Multiple pregnancy, Twins, Preterm birth, Perinatal outcomes

## Abstract

**Objective:**

Multiple pregnancies carry an increased risk of maternal and perinatal complications, notably prematurity. Few studies have evaluated the risk factors for preterm births in multiple pregnancies within the Thai population. This study aims to ascertain maternal and perinatal outcomes and identify factors linked to preterm births in multiple pregnancies.

**Methods:**

This study was carried out at Khon Kaen University, Faculty of Medicine, Department of Obstetrics and Gynecology in Thailand. We reviewed the medical records of women with multiple pregnancies who delivered at a gestational age of more than 20 weeks between January 1, 2012 and December 31, 2021. We excluded patients with incomplete data or those for whom data were missing.

**Results:**

Out of 21,400 pregnancies, 427 were multiple pregnancies, constituting approximately 1.99%. Over the ten-year period, 269 multiple pregnancies (65.1%) resulted in preterm births. Of these, 173 (64.3%) were monochorionic twins, and 96 (35.7%) were dichorionic twins. Monochorionic twins had a notably higher rate of preterm delivery (AOR, 2.06; 95%CI 1.29—3.30). Vaginal delivery was observed in 7.9% of the cases, while cesarean sections were performed for both twins in 91.5% of cases. In 0.5% of the cases, only the second twin was delivered by cesarean section. In terms of neonatal outcomes, 160 infants (19.4%) weighed less than 1,500 g at birth, and there were 78 perinatal deaths (9.4%). Birth asphyxia was noted in 97 cases (20.2%) among monochorionic twins and in 28 cases (8.1%) for dichorionic twins.

**Conclusion:**

The prevalence of multiple pregnancies was 1.99%, with 65.1% resulting in preterm births. Neonatal complications were notably more frequent in monochorionic twins. Monochorionic placenta and antepartum complications emerged as significant risk factors for preterm birth.

## Introduction

Since 1980, there has been a 76% increase in multiple pregnancy (defined as a pregnancy with two or more fetuses such as triplets or quadruplets) – a trend that continues to this day [1]. These pregnancies are deemed high-risk, often leading to complications for both the mother and the fetus. The severity of complications tends to increase with the number of fetuses present [1–3]. For mothers carrying twins, common complications include high blood pressure, pre-eclampsia, gestational diabetes, anemia, postpartum hemorrhage, cesarean section, and postpartum depression [4–10]. Common complications in fetuses include growth restriction, premature birth, and death, which is five times higher in twin than in singleton pregnancies. Notably, as the number of fetuses increases, so does the risk of fetal death—with twins, triplets, and quadruplets facing 4, 12, and 26 times the risk, respectively [11]. In particular, preterm delivery exacerbates the potential for significant neonatal morbidity and mortality [12–14].

Preterm birth refers to any birth occurring before 37 weeks of gestation. A notable risk factor for preterm birth is chorionicity [15], with twins born from monochorionic pregnancies exhibiting higher rates of neonatal morbidity and mortality [16]. The causes of premature birth in multiple pregnancies are complex and multifactorial. Factors that contribute to increased risk of premature birth include 1) uterine overstretching, cervical changes, and increased pressure on the cervix; 2) placental complications that necessitate early delivery (e.g., placental abruption or placenta previa); 3) maternal conditions such as gestational diabetes, preeclampsia, which are more common in multiple pregnancies; and 4) fetal conditions such as fetal growth restriction and twin-specific complications that arise during the pregnancy, which can result in an early delivery [17, 18].

Thus, multiple pregnancies present an elevated risk for maternal and perinatal complications, particularly prematurity. Despite the obvious risks, few studies have explored the general factors leading to preterm births in multiple pregnancies. However, other specific factors related to multiple pregnancies, such as method of conception, preeclampsia, complications, comorbidities, and other specific conditions, should be explored. This study aimed to determine the prevalence of preterm birth in such pregnancies, to understand maternal and perinatal outcomes, and to identify associated risk factors.

## Material and methods

This retrospective cohort study was conducted at Khon Kaen University’s Srinagarind Hospital, a tertiary care facility serving northeast Thailand. We included women with multiple pregnancies who delivered at a gestational age beyond 20 weeks from January 1, 2012, to December 31, 2021. Medical records were thoroughly reviewed, and cases with incomplete or missing data were excluded.

Data collected included baseline characteristics, obstetric data, diagnosis, management, and perinatal outcomes such as age, pre-pregnancy BMI, weight gain during pregnancy, underlying disease, pregnancy conception, chorionicity, twin complications, steroid administration, history of preterm birth, gestational age at delivery, delivery route, antepartum complications (e.g., placenta previa, abruption, placenta accreta syndrome, prolapsed cord, vasa previa, chorioamnionitis, pulmonary edema, and congestive heart failure), intrapartum and postpartum complications, and perinatal outcomes.

We compared various maternal complications (e.g., preterm birth, premature rupture of membranes, delivery mode, antepartum complications, pregnancy-induced hypertension, gestational diabetes, postpartum hemorrhage, and ICU admission), and perinatal outcomes (e.g., complicated conditions, birth asphyxia with an Apgar score at 5 min < 7, weight discordance between fetuses ≥ 20%, very low birth weight < 1,500 g, perinatal death, and NICU admission) between monochorionic and dichorionic twins. Additionally, we examined associated risk factors for preterm birth in twin pregnancies.

Statistical analysis was performed using STATA version 10.1 (StataCorp LP, College Station, TX, USA). For continuous variables, Student's t-test was applied to normally distributed data and the Mann–Whitney U-test to skewed data. Chi-square or Fisher’s exact tests were employed for categorical variables. The prevalence of multiple pregnancies was documented per 100 deliveries and determined at a 95% confidence interval (CI). Secondary outcomes were presented as percentages, means with standard deviations, medians with interquartile ranges, and adjusted odds ratios at 95% CI. We compared characteristics between monochorionic and dichorionic twins. A *p*-value of < 0.05 was deemed statistically significant. The required sample size was determined to be 381 twin pregnancies, based on a preterm birth prevalence in twin pregnancies of 55 per 100 deliveries [13, 15, 19].

The study protocol was reviewed and approved by the Khon Kaen University Ethics Committee for Human Research (HE651085) and registered in the Thai Clinical Trial Registry (TCTR20230826012).

## Results

**Fig. 1:**
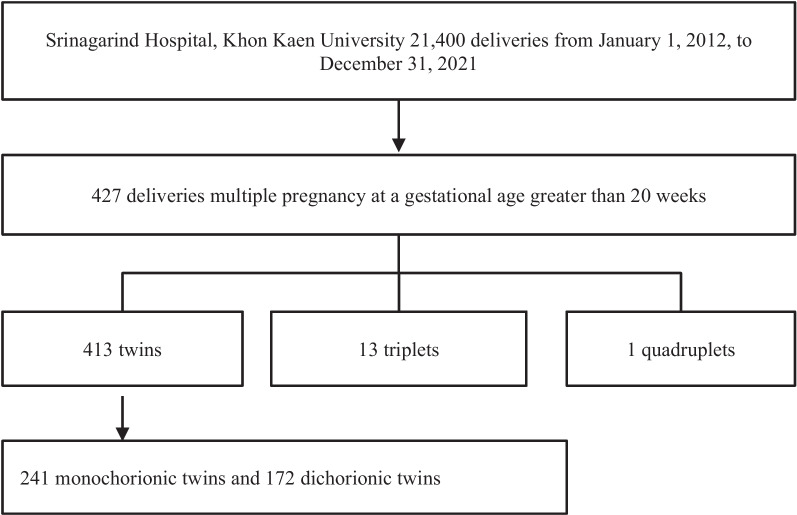
Flow diagram of pregnant women

Of the 21,400 deliveries during the study period, 427 were identified as multiple pregnancies, as shown in Fig. 1. The overall prevalence of multiple pregnancies was 1.99% (95% CI 1.81, 2.19) over the ten-year study duration. The breakdown included 413 twins, 13 triplets, and one set of quadruplets. Table 1 details the baseline characteristics of these multiple pregnancies. Among these, 100 women (23.42%) were aged 35 years or older. Nearly half (46.14%) were nulliparous, 11 (2.58%) had a prior preterm birth, and 71 (16.63%) conceived via IVF. The most prevalent underlying medical condition was anemia, affecting 64 women (14.9%). In the 13 cases of triplets, the mean gestational age at birth was 34 complete weeks, with a mean birth weight of 1,841 g among the 30 live-born fetuses. There were 7 stillbirths and 2 intrapartum deaths among the fetuses. In one case of quadruplets, delivery occurred at 32 completed weeks, and the birth weights were 1,590 g, 1,500 g, and 1,325 g, with no preterm birth complications.

**Table 1:** Baseline characteristics of women with multiple pregnancies

Characteristics	*N* = 427
Age – years ^a^	30.1 ± 5.9
Age group—n (%)	
• < 20 years	21 (4.9)
• 20–34 years	306 (71.7)
• ≥ 35 years	100 (23.4)
Mean pre-pregnancy BMI (kg/m^2^)	21.65 (3.9)
BMI category – n (%)	
• < 18.5 kg/m^2^	87 (20.3)
• 18.5 – 24.9 kg/m^2^	264 (61.8)
• 25 – 29.9 kg/m^2^	62 (14.6)
• ≥ 30 kg/m^2^	14 (3.3)
Mean weight gain during pregnancy – kg ^a^	15.2 ± 5.9
Nulliparous – n (%)	197 (46.1)
History of preterm birth – n (%)	11 (2.6)
In vitro fertilization method—n (%)	71 (16.6)
Underlying medical conditions	
• Anemia—n (%)	64 (14.9)
• Chronic hypertension—n (%)	6 (1.4)
• Overt diabetes mellitus—n (%)	2 (0.5)
• Hypothyroidism—n (%)	7 (1.6)
• Hyperthyroidism—n (%)	7 (1.6)
• Connective tissue diseases—n (%)^b^	1 (0.2)
Twins—n (%)	413 (96.7)
• Dichorionic diamniotic– n (%)	172 (41.65)
• Monochorionic diamniotic– n (%)	219 (53.03)
• Monochorionic monoamniotic– n (%)	22 (5.15)
Triplets—n (%)	13 (3.04)
Quadruplets—n (%)	1 (0.23)

^a^ Values are mean ± SD

^b^ connective tissue diseases included systemic lupus erythematosus and antiphospholipid syndrome

Table 2 presents the diagnosis and delivery characteristics of monochorionic and dichorionic twin pregnancies. The average gestational age at delivery for monochorionic twins was significantly lower than that of dichorionic twins [0.87 (0.81–0.93), *p* < 0.001], irrespective of whether the gestational age was under 28 weeks, between 28–34 weeks, or less than 37 weeks. Among the monochorionic pregnancies, 19 fetuses (7.9%) had structural abnormalities (acardiac twins *n* = 6, anencephaly *n* = 6, conjoined twins *n* = 3, omphalocele *n* = 2, congenital cystic adenomatoid malformation *n* = 1, encephalocele *n* = 1, and multiple anomalies *n* = 1), compared to 3 fetuses (3%) in the dichorionic group (omphalocele, gastroschisis, and spina bifida), indicating a significant difference [5.55 (1.54, 19.95), *p* = 0.009]. The dichorionic group had a notably higher rate of IVF conceptions compared to the monochorionic group [0.12 (0.06–0.25), *p* < 0.001]. Spontaneous preterm births were more frequent in monochorionic twins (52.3%) than their dichorionic counterparts (43.6%), with the former having a significantly higher rate of preterm births [2.04 (1.32, 3.14), *p* < 0.001]. While trying to inhibit preterm labor, the monochorionic group had a lower (albeit not significantly so) success rate (11.8%) compared to the dichorionic group (25.4%). For delivery methods, 7.9% were vaginal, whereas cesarean sections were performed in 91.5% of cases. Two cases (0.5%) in the monochorionic group underwent a cesarean section for the second twin due to cord prolapse and an abnormal twin presentation. The primary reason for cesarean sections was multiple pregnancy.

**Table 2:** Diagnosis and delivery characteristics of twin pregnancies

Characteristic	Monochorionic twins (*N* = 241)	Dichorionic twins (*N* = 172)	Adjusted OR (95% CI)	*P*-value
Structural abnormality – n (%)	19 (7.9)	3 (1.7)	5.55 (1.54, 19.95)	0.009
Chromosome abnormality – n (%)	0	1	NA	
In vitro fertilization conception– n (%)	11 (4.6)	58 (33.7)	0.12 (0.06, 0.25)	< 0.001
Mean gestational age at delivery	34.13 (4.10)	35.72 (2.61)	0.87 (0.81, 0.93)	< 0.001
• < 28 weeks	21 (8.7)	04 (2.3)		
• 28 – < 34 weeks	57 (23.7)	24 (14.0)		
• 34—< 37 weeks	95 (39.4)	68 (39.5)		
• ≥ 37 weeks	68 (28.2)	76 (44.2)		
Preterm birth	173 (71.8)	96 (55.8)	2.04 (1.32, 3.14)	0.001
• Spontaneous preterm birth – n (%)	126 (52.3)	75 (43.6)		
• Induced preterm birth – n (%)	47 (19.5)	21 (12.2)		
Preterm labor with inhibition	93 (38.6)	59 (34.3)	0.93 (0.67, 1.31)	0.693
• Failed – n (%)	82 (88.2)	44 (74.6)		
• Successful – n (%)	11 (11.8)	15 (25.4)		
Preterm PROM	41 (17.3)	35 (20.5)	0.80 (0.47, 1.35)	0.401
Dexamethasone				0.624
• Partial (≤ 3 dose) – n (%)	19 (7.9)	10 (5.9)	1.34 (0.57, 3.16)	
• Complete – n (%)	76 (31.7)	46 (27.1)	1.28 (0.80, 2.03)	
• Rescue course – n (%)	8 (3.3)	3 (1.8)	1.76 (0.44, 6.95)	
Mode of delivery				0.092
• Previous CS – n (%)	32 (13.3)	19 (11.1)	1	
• Primary CS – n (%)	182 (75.5)	145 (84.3)	0.58 (0.29, 1.14)	
• Vaginal delivery and CS in second twin – n (%)	2 (0.81)	0 (0.00)	NA	
• Vaginal delivery – n (%)	25 (10.4)	8 (4.7)	1.21 (0.43, 3.42)	
Indication for cesarean section				0.271
• Multiple pregnancy – n (%)	110 (47.3)	83 (50.6)	0.56 (0.24, 1.31)	
• Abnormal presentation in first twin – n (%)	44 (18.3)	43 (25.0)	0.45 (0.18, 1.12)	
• Non-reassuring fetal status – n (%)	19 (7.9)	8 (4.7)	1.06 (0.32, 3.45)	
• Failed induction – n (%)	5 (2.1)	1 (0.6)	2.07 (0.20, 21.61)	
• Antepartum hemorrhage – n (%)	3 (1.2)	6 (3.5)	0.41 (0.08, 2.20)	
• Preeclampsia – n (%)	2 (0.8)	3 (1.7)	0.36 (0.05, 2.63)	
• Prolapsed cord – n (%)	1 (0.41)	0 (0.00)	NA	

*CS* cesarean section, antepartum hemorrhage; placenta previa, placental abruption and placenta accreta. Adjusted by age, parity, pre-pregnancy BMI and underlying medical conditions

Table 3 compares antepartum and intrapartum complications, along with maternal outcomes, between monochorionic and dichorionic twins. The monochorionic group exhibited a significantly higher rate of twin-related complications compared to the dichorionic group [2.88 (1.83, 4.54), *p* < 0.001].

**Table 3:** Antenatal, intrapartum complication and maternal outcomes in twin pregnancy

**Characteristic**	**Monochorionic twins (*N*=241)**	**Dichorionic twins (*N*=172)**	**Adjusted OR (95% CI)**	***P*-value**
Antepartum complications – total n (%)	9 (3.7)	11 (6.4)	0.74 (0.28, 1.94)	0.536
• Placenta previa – n (%)	2 (0.8)	5 (2.9)		
• Abruption – n (%)	2 (0.8)	0 (0.0)		
• Placenta accreta syndrome – n (%)	1 (0.4)	1 (0.6)		
• Prolapsed cord – n (%)	1 (0.4)	1 (0.6)		
• Vasa previa – n (%)	0 (0.0)	0 (0.0)		
• Chorioamnionitis – n(%)	2 (0.8)	0 (0.0)		
• Pulmonary edema – n (%)	1 (0.4)	3 (1.7)		
• Congestive heart failure – n (%)	0 (0)	1 (0.6)		
Twin-related complications – total n (%)	114 (47.3)	40 (23.3)	2.88 (1.83, 4.54)	<0.001
• Discordant twin – n (%)	92 (38.2)	36 (20.9)		
• Selected fetal growth restriction – n (%)	45 (18.7)	10 (5.8)		
• Twin-to-twin transfusion syndrome (TTTS) – n (%)	30 (12.5)	0 (0.0)		
• Acardiac twins – n (%)	6 (2.5)	0 (0.0)		
• Single fetal demise – n (%)	24 (10.0)	6 (3.5)		
• Twin anemia-polycythemia sequence (TAPS)	1 (0.4)	0 (0)		
• Twin reversed arterial perfusion (TRAP sequence)	3 (1.2)	0 (0)		
• Conjoined	3 (1.2)	0 (0)		
Pregnancy induced hypertension – total n (%)	25 (10.4)	20 (11.6)	0.83 (0.42, 1.64)	0.596
• Chronic hypertension	3 (1.2)	3 (1.7)		
• Gestational hypertension	5 (2.1)	1 (0.6)		
• Preeclampsia without severe features	8 (3.3)	6 (3.5)		
• Preeclampsia with severe features	8 (3.3)	10 (5.8)		
• Eclampsia	1 (0.4)	0 (0.0)		
Gestational diabetes mellitus – total n (%)	19 (7.9)	18 (10.5)	1.08 (0.49, 2.41)	0.845
• Insulin control – n (%)	4 (1.7)	6 (3.5)		
Postpartum hemorrhage	51 (21.2)	49 (28.5)	0.73 (0.45, 1.17)	0.187
Intraoperative intervention – total n (%)	13 (5.4)	6 (3.5)	2.73 (0.92, 8.05)	0.069
ICU admission – n (%)	0 (0.0)	6 (3.5)	NA	
Blood transfusion – n (%)	2 (0.8)	2 (1.2)	1.36 (0.18, 10.38)	0.766

Postpartum hemorrhage: blood loss ≥ 500 mL. Intraoperative intervention: hysterectomy, bowel and bladder repair. Adjusted by age, parity, pre-pregnancy BMI and underlying medical conditions

Neonatal outcomes in monochorionic and dichorionic twin pregnancies are compared in Table 4. Birth asphyxia, defined by an APGAR score of < 7 at 5 min after birth, was notably higher in the monochorionic group [2.91 (1.64, 5.16), *p* < 0.001]. Additionally, the average birth weight was significantly lower in the monochorionic group [0.41 (0.29, 0.56), *p* < 0.001]. Very low birth weight (BW < 1,500 g) was more common in the monochorionic group (26.8% vs. 9% in the dichorionic group). Discordant weight exceeding 20% was significantly more prevalent in the monochorionic group [2.25 (1.41, 3.59), *p* < 0.001]. Notably, the monochorionic group had a significantly higher rate of perinatal death (*p* < 0.001).

**Table 4:** Neonatal outcomes in twin pregnancy

Characteristic	Monochorionic twins (*N* = 482)	Dichorionic twins (*N* = 344)	Adjusted OR (95% CI)	*P*-value
Birth asphyxia—n (%)	97 (20.2)	28 (8.1)	2.91 (1.64, 5.16)	< 0.001
Average of mean birth weight – g	1.87 (.74)	2.27 (.58)	0.41 (0.29, 0.56)	< 0.001
• < 1,500 g—n (%)	129 (26.8)	31 (9.0)		
• 1,500–2,000 g—n (%)	108 (22.5)	49 (14.2)		
• 2,001–2,500 g—n (%)	141 (29.3)	141 (41.0)		
• ≥ 2,500—n (%)	98 (20.4)	122 (35.5)		
Significant wight discordance—%	184 (38.2)	72 (20.9)	2.25 (1.41, 3.59)	0.001
Death				< 0.001
• Stillbirth—n (%)	47 (9.8)	6 (1.7)	6.70 (2.78, 16.16)	
• Intrapartum death—n (%)	13 (2.7)	0 (0.0)	NA	
• Neonatal death – n (%)	9 (1.9)	3 (0.9)	3.30 (0.61, 17.76)	
Neonatal resuscitation—n (%)	96 (20.0)	64 (18.6)	1.08 (0.67, 1.74)	0.745
NICU admission—n (%)	114 (23.7)	69 (20.1)	1.21 (0.77, 1.90)	0.419

Significant wight discordance: weight discordance between fetuses ≥ 20%. Neonatal resuscitation: neonates requiring continuous positive airway pressure, positive pressure ventilation, chest compressions, or endotracheal intubation. Adjusted by age, parity, pre-pregnancy BMI, and underlying medical conditions

Table 5 shows factors associated with preterm birth in twin pregnancies. Monochorionic twins exhibited a significantly higher rate of preterm birth [2.08 (1.30, 3.34), *p* = 0.002]. For those who delivered preterm, a significantly higher number of antepartum complications were observed (such as placenta previa, abruptio placentae, placenta accreta syndrome, cord prolapse, vasa previa, chorioamnionitis, and pulmonary edema) compared to term deliveries [12.99 (1.69, 9.58), *p* = 0.014].

**Table 5:** Factors associated with preterm delivery in twin pregnancies

Characteristic	Term delivery (*N* = 144)	Preterm delivery (*N* = 269)	Adjusted OR (95% CI)	*P*-value
Monochorionic twin	68 (47.2)	173 (64.3)	2.08 (1.30, 3.34)	0.002
History of preterm birth	1 (0.7)	10 (3.7)	6.20 (0.76, 50.29)	0.087
Antepartum complications	1 (0.7)	19 (7.1)	12.99 (1.69, 9.58)	0.014
IVF conception	27 (18.9)	42 (15.6)	1.08 (0.59, 1.98)	0.810
Complicated twin pregnancy	32 (22.2)	80 (29.7)	1.29 (0.78, 2.13)	0.328
Preeclampsia	10 (6.9)	22 (8.2)	1.19 (0.53, 2.67)	0.674

Advanced maternal age: age ≥ 35 years old, Preeclampsia includes both severe, non-severe feature, HELLP and eclampsia. Adjusted by age, parity, pre-pregnancy BMI, and underlying medical conditions

## Discussion

This research sheds light on the maternal and neonatal outcomes of twin pregnancies and examines the risk factors associated with preterm birth in these pregnancies in a Thai population. As per the findings of this population-based research, the overall prevalence of multiple pregnancies was 1.99% of all deliveries. The incidence of preterm births in twin pregnancies was 65.1%, with monochorionic twins exhibiting significantly higher rates of preterm births and neonatal complications. The average gestational age at which monochorionic twins were delivered was notably earlier compared to dichorionic twins. Our findings further indicate that monochorionicity is linked with a heightened risk of spontaneous preterm birth and a greater risk of complications among twins, as evidenced by higher rates of birth asphyxia, reduced average birth weights, discordancy between twins, and perinatal deaths.

The past decade has seen a rise in the prevalence of twin deliveries. The recorded rate of 1.99 per 100 live births in this study is higher than figures reported in previous Thai studies conducted between 2004–2009 but align well with studies from the decade of the current study [13–15, 18–21]. The incidence of preterm birth in twin pregnancies found in this research was comparable to prior studies [13, 15, 19, 20]. Drawing from the work of Santana et al., our study echoes the finding that the primary culprit behind preterm births in twin pregnancies is the spontaneous onset of preterm labor, accounting for 74.7% of the preterm twin births recorded here [21]. Other studies, like those by Oger et al. and Feng et al., corroborate our observations that neonates from monochorionic twin pregnancies face heightened morbidities. These include very low birth weight (less than 1,500 g), Apgar scores below 7 at 1 min, and a significantly increased perinatal mortality rate [15, 16]. We also observed a greater likelihood of cesarean section in multiple pregnancies. Our study found that 88.5% of such pregnancies ended in cesarean section, with 76.9% of these being primary cesarean deliveries. These figures align with the findings of prior research [13–15, 21]. To sum up, we found that monochorionicity and the presence of antepartum complications were factors associated with preterm birth in multiple pregnancies.

Factors associated with preterm birth in twin pregnancies, such as monochorionic twins or a history of previous preterm birth, may pose significant challenges in terms of prevention. However, in cases of antepartum complications, such as chorioamnionitis, close monitoring, early diagnosis, and appropriate management can potentially extend gestation and facilitate referral for optimal care at a tertiary care facility. Preeclampsia, including severe features (pulmonary edema and congestive heart failure), can be mitigated by the administration of low-dose aspirin to reduce the risk of preeclampsia, a major cause of indicated preterm birth in both singleton and twin pregnancies.

Placenta accreta syndrome is frequently encountered in individuals with a history of prior cesarean sections. Therefore, if such a history exists, physicians should consider single embryo transfer in cases of in vitro fertilization to prevent multiple pregnancies.

In specific conditions involving complicated twin pregnancies (e.g., discordant twins, selective fetal growth restriction, twin-to-twin transfusion syndrome), as well as cases of antepartum hemorrhage (e.g., placenta previa and placental abruption), prompt diagnosis by specialists and close monitoring for potential early delivery can be challenging. Consideration should be given to the administration of steroids to enhance lung maturity and minimize complications associated with preterm birth. Increasing surveillance and preparing healthcare facilities for the management of premature delivery is crucial to ensure optimal care for multiple pregnancies.

Our study found that pregnancies with triplets carried a high incidence of antenatal stillbirth. This is a matter that warrants further study, both in terms of causes and prevention. There was only one case of quadruplet pregnancy, and the outcome was considered very favorable. Nevertheless, further research examining data from multiple institutions is necessary.

### Strengths and limitations

While this retrospective study provided valuable insights, certain limitations arose due to incomplete records. Specifically, the paucity of data on triplets and quadruplets precluded comprehensive analysis. However, the data extraction from the electronic medical records benefited from the robust sample size. Notably, this study had the largest cohort of women with multiple pregnancies in Thailand.

### Implications and future research

We assessed the epidemiology, maternal characteristics, and perinatal outcomes of multiple pregnancies. Further large, high-quality, and multi-center studies are required to assess the spectrum of disease and maternal and perinatal outcomes including factors associated with preterm birth and long-term implications. Any protocols aimed at predicting and preventing complications in multiple pregnancies in Thailand would greatly benefit from the clinical data from our population.

## Conclusions

The prevalence of multiple pregnancy in this study was 427 out of 21,400 pregnancies (1.99%). Neonatal complications were significantly higher in monochorionic twins. Both the presence of a monochorionic placenta and antepartum complications were significant risk factors for preterm birth.

## Data Availability

The data used to support the findings of this study are available from the corresponding author upon request.
